# Splice-modulating antisense oligonucleotides targeting a pathogenic intronic variant in adult polyglucosan body disease correct mis-splicing and restore enzyme activity in patient cells

**DOI:** 10.1093/nar/gkaf658

**Published:** 2025-07-16

**Authors:** Ria Thomas, Emily Miyoshi, Hasan O Akman, Sophie H R Storz, Joy Goffena, Julia Pytte, Danny E Miller, Konstantina Skourti-Stathaki, Stanley T Crooke

**Affiliations:** n-Lorem Foundation, Carlsbad, CA 92010, United States; n-Lorem Foundation, Carlsbad, CA 92010, United States; Department of Neurology, Division of Neuromuscular Medicine, Columbia University Irving Medical Center, New York, NY 10032, United States; Department of Pediatrics, Department of Laboratory Medicine and Pathology, Brotman Baty Institute for Precision Medicine, University of Washington, Seattle, WA 98195, United States; Department of Pediatrics, Department of Laboratory Medicine and Pathology, Brotman Baty Institute for Precision Medicine, University of Washington, Seattle, WA 98195, United States; n-Lorem Foundation, Carlsbad, CA 92010, United States; Department of Pediatrics, Department of Laboratory Medicine and Pathology, Brotman Baty Institute for Precision Medicine, University of Washington, Seattle, WA 98195, United States; n-Lorem Foundation, Carlsbad, CA 92010, United States; n-Lorem Foundation, Carlsbad, CA 92010, United States

## Abstract

Adult polyglucosan body disease (APBD) is a rare, adult-onset neurodegenerative disorder caused by loss-of-function variants in the glycogen branching enzyme (*GBE1*) gene, essential for glycogen biosynthesis. The second most common pathogenic mutation in APBD (c.2053-3358_2053-3350delinsTGTTTTTTACATGACAGGT) is a deep intronic deletion insertion (indel) variant creating an ectopic splice acceptor site, resulting in a mutant transcript with a pseudoexon encoding an unstable truncated protein. Such mutations can be effectively targeted with splice-modulating antisense oligonucleotides (ASOs) to restore normal splicing. Here, we characterized the indel in-depth using long-read sequencing techniques and discovered several new features of the mutant transcript. The indel sequence varies from the previously identified sequence by a nucleotide, and the usage of the ectopic splice site results in two mutant isoforms, both of which are targets of cellular nonsense-mediated decay. High-throughput screening in patient-derived fibroblasts identified multiple lead candidates that effectively blocked the ectopic splice site and increased the canonical *GBE1* transcript and protein. Functional analysis confirmed that treatment with the lead ASOs significantly improved GBE1 enzyme activity in patient cells, validating their therapeutic potential. Taken together, our data demonstrate the successful discovery of ASOs that correct mis-splicing, thus offering a promising treatment for a subset of APBD patients.

## Graphical Abstract

**Graphical Abstract:**
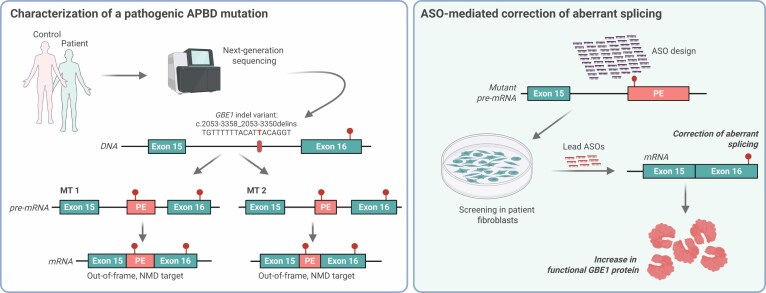

## Introduction

Adult polyglucosan body disease (APBD) is a rare, adult-onset, progressive neurodegenerative disorder characterized by a spectrum of clinical manifestations, including peripheral neuropathy, neurogenic bladder, fatigue, muscle weakness and spasticity, and autonomic dysfunction [1, 2]. Some patients also exhibit cognitive impairment and visual dysfunction [3–5]. This autosomal recessive condition is caused by mutations in the *GBE1* gene, which encodes the glycogen branching enzyme essential for glycogen biosynthesis. The resulting deficiency in GBE1 activity leads to the accumulation of abnormally structured, insoluble glycogen, manifesting as polyglucosan bodies (PBs) in various tissues [5–7]. The deposition of PBs in neurons of both the central and peripheral nervous systems likely blocks the axons, potentially contributing to the neurological symptoms observed in affected individuals [8]. While multiple therapeutic approaches aimed at decreasing GBE1 substrate levels by targeting glycogen synthase 1 [9–11], enhancing GBE1 enzyme activity [12], and promoting lysosomal degradation of PBs [13] are in preclinical and clinical development, there are currently no effective disease-modifying therapies available for APBD.

Presently, there are over 200 genetically confirmed cases of APBD worldwide, with a significant majority, ∼85.1%, of affected individuals in the United States belonging to the Ashkenazi Jewish population [14, 15]. Due to its rarity and symptom overlap with other neurological disorders, APBD is often misdiagnosed and consequently underestimated [16]. Recent estimates indicate that the true prevalence may be much higher, with as many as 3400 to 6300 individuals affected within the Ashkenazi Jewish community in the U.S. alone [17]. The second most common pathogenic mutation in the *GBE1* gene among this population is a deep intronic deletion-insertion (indel) variant [NM_000158.4(GBE1):c.2053-3358_2053-3350delinsTGTTTTTTACATGACAGGT] [15, 18]. This variant consists of a 9-nucleotide deletion and a 19-nucleotide insertion located deep within intron 15. This genetic alteration activates an ectopic splice acceptor site within the indel, resulting in the formation of a pseudoexon in intron 15 of the mutant (MT) *GBE1* transcript. This pseudoexon, identified as the last exon of the MT transcript, contains a premature termination codon (PTC). Because the PTC is in the final exon, the MT transcript escapes degradation by the cellular nonsense-mediated decay (NMD) pathway. The resultant truncated MT protein produced is unstable and dysfunctional [18]. While the initiation point of the pseudoexon in the MT transcript has been identified, the precise 3′ terminus as well as the nature of the MT transcript remain to be determined.

Antisense oligonucleotides (ASOs) are short, single-stranded, chemically modified oligonucleotides that bind to complementary RNA sequences by Watson–Crick base pairing to modulate gene expression through different post-hybridization mechanisms (RNAse H1-mediated degradation or steric interference-mediated up- or downregulation) [19]. Splice-modulating ASOs are a type of steric interference ASOs designed to alter splicing by binding to a specific precursor messenger RNA (pre-mRNA) sequence to interfere with the recognition and binding of splicing factors. Deep intronic pathogenic mutations that result in the inclusion of a pseudoexon, while maintaining intact canonical splice sites, are ideal candidates for therapeutic targeting by splice-modulating ASOs, and this has been validated in patient-derived cells from multiple diseases [20–29].

In this manuscript, we report an in-depth characterization of the *GBE1* indel mutation and the identification of effective splice-modulating ASOs capable of restoring functional GBE1 protein in patient cells. Utilizing advanced whole genome and transcriptome sequencing technologies, we provide a comprehensive analysis of the mutation and uncover multiple previously unknown features of the MT transcript. High-throughput screening in patient cells identified several lead ASOs that effectively blocked the ectopic splice site, resulting in increased levels of functional GBE1 enzyme, demonstrating therapeutic potential. These results underscore the potential of ASOs as a viable therapeutic option for addressing the underlying genetic defects in a subset of APBD patients.

## Materials and methods

### Cell culture

The three apparently healthy subject-derived fibroblast cell lines (HS 1 - GM02936, HS 2 - GM03440, HS 3 - GM03529) were obtained from the NIGMS Human Genetic Cell Repository at the Coriell Institute for Medical Research (Camden, NJ). Fibroblasts from patients 1, 2, and 4 were provided by Dr. Hirano Michio at the Columbia University Medical Center, while fibroblasts from patient 3 were received from Dr. Niccolo Mencacci at Northwestern University. HeLa cells were a kind gift from Ionis Pharmaceuticals (Carlsbad, CA), and Bjab cells (#ACC 757) were purchased from the DSMZ cell repository (Braunschweig, Germany).

Fibroblasts and HeLa cells were cultured in Dulbecco’s modified Eagle’s medium high glucose media (Gibco, #11965-084) supplemented with 10% fetal bovine serum (FBS) (Gibco, #26140-079) and 1% penicillin-streptomycin (Cytiva, #SV30010). Cultures were grown in T75 or T175 flasks and were passaged when 80%–90% confluent using 0.25% trypsin ethylenediaminetetraacetic acid (EDTA) (Gibco, #25200-056). Fibroblasts used for experiments were not kept in culture beyond 10 passages. Bjab cells were grown in suspension culture in Roswell Park Memorial Institute (RPMI) 1640 medium (Gibco, #11875-093) supplemented with 20% heat-inactivated FBS (Gibco, #A52568-01) and 1% penicillin-streptomycin (Cytiva, #SV30010) solution. Cells were maintained at a density of 3–9 × 10^5^ cells/ml and were passaged when they exceeded the recommended culture density. All cells were kept in an incubator at 37°C and 5% CO_2_.

### Cycloheximide treatment

For the cycloheximide treatment, fibroblasts were plated at a density of 45000 cells/well in 96-well plates using regular growth medium. Six hours after plating, the medium was replaced with media containing varying concentrations of cycloheximide (Thermo Fisher, #J66004). Media with DMSO was used as a vehicle control. Sixteen hours post-treatment, the cells were lysed, and RNA was isolated as described below.

### siRNA treatment

For small interfering RNA (siRNA) treatment, fibroblasts were plated at 90000 cells/well in 12-well plates, and HeLa cells were plated at 40000 cells/well in 24-well plates. The following day, siRNAs targeting *GBE1* (Horizon Discovery, #L-011039-00-0005) and a nontargeting control (Horizon Discovery, #D-001810-10) were transfected at final concentrations of 10 and 30 nM using Lipofectamine RNAiMAX transfection reagent (Thermo Fisher, #13778075) according to the manufacturer’s protocol. The medium was replaced with fresh normal growth medium the next day. Three days post transfection, the cells were lysed for RNA and protein analyses as detailed below.

### ASO treatment

All ASOs used in this study were manufactured by Integrated DNA Technologies (IDT). They were reconstituted in nuclease-free water at concentrations of 1 mM for the Bjab assay, 400 μM for transcript analysis, and 800 μM for protein and enzyme activity assays. The reconstituted ASOs were heated at 55°C for 10 min, mixed well, and stored at −80°C until use. For ASO treatment, 7.5 μl of stock ASOs were added to each well of a 96-well disposable electroporation plate (BTX, #45-0452), followed by the addition of fibroblasts at a density of 40000 cells/well in 142.5 μl of medium. Cells were electroporated using a single pulse at 260 V for 6 milliseconds with the ECM 830 High Throughput Electroporation System (BTX, #45-0666). After electroporation, the entire cell suspension (150 μl) from each well was transferred to 96-well flat-bottom tissue culture-treated plates (Corning, #3595) along with an additional 50 μl of medium. The plates were then incubated at 37°C, and cells were harvested for RNA isolation 18–22 h later. For protein assays, cells were electroporated using the same protocol. However, seven wells treated with the same ASO were pooled together and plated into one well of a six-well plate with an additional 1 ml of medium. Cells from the six-well plates were harvested four days later for protein and enzyme activity assays as described below.

### RNA isolation and qRT-PCR

RNA extraction was performed using a protocol adapted from Cytiva (https://cdn.cytivalifesciences.com/api/public/content/u2QmauFrs0yDr4tB8EUABg-pdf) with modifications to fit a 384-well format. Samples in 96-well plates were washed once with 1× DPBS (without Ca^2+^ and Mg^2+^), lysed in 40 μl GTC lysis buffer (Invitrogen, #15577018), and mixed thoroughly on a plate shaker for 3 min. An equal volume of 70% ethanol (Sigma, #T03818) was added to each well, mixed thoroughly, and samples were transferred to a Pall AcroPrep 384-well filter plate (Pall, #5072-N). The rest of the protocol followed the original with adjustments to the volumes of RNA wash buffer (first wash: 42.5 μl instead of 170 μl; second wash: 62.5 μl instead of 200 μl), DNase I buffer (20 μl instead of 80 μl), and GTC wash buffer (42.5 μl instead of 170 μl) used during extraction. Final elution was performed in 50 μl nuclease-free water in a 384-well clear tissue culture treated plates (Falcon, #353961). Samples were stored at −80°C until ready to be used for downstream assays such as quantitative reverse transcription polymerase chain reaction (qRT-PCR) and RNA sequencing (RNA-seq).

qRT-PCR reactions were performed using AgPath-ID One-Step RT-PCR reagent (Thermo Fisher, #4387391) according to the manufacturer’s instructions. Each 10 μl reaction consisted of 1× RT-PCR buffer, 1× RT-PCR enzyme mix, 4 μl sample RNA, 1 μM each of reverse and forward primers, and 0.2 μM of probe. Reverse transcription was carried out at 45°C for 10 min, followed by 95°C for 10 min. The PCR cycles were then conducted at 95°C for 15 s and 60°C for 45 s, repeated for 40 cycles. qPCR reaction was run on QuantStudio 7 Pro (Thermo Fisher) and analysis was performed using the 2^−ΔΔCT^ method [30]. *PPIA* or *GAPDH* were used as housekeeping genes to normalize mRNA levels in each sample. All primers and probes used in this study were ordered from IDT. The primer probe sequences used for the detection of the *GBE1* transcripts are given below. *GAPDH* was amplified using a commercial assay (IDT, #Hs.PT.39a.22214836) and the primer probe set for *PPIA* (sequence not shown) was designed by Ionis Pharmaceuticals.

#### GBE1

WT and MT forward primer: 5′-CAG ATG CAG CGG AAT ATG GA-3′

WT reverse primer: 5′-GAG GGC CAC TCT GCT TG-3′

MT reverse primer: 5′-ATG TTG GTA AGG CTG GTC TC-3′

WT and MT probe: 5′-AGA CTG GAC CAC AGC ACT GAC TTT-3′ with 3′ Iowa black, 5′ 6-FAM and internal ZEN modifications

### Whole genome sequencing

DNA for sequencing was isolated from whole blood using the Qiagen Puregene kit following the manufacturer’s instructions. Targeted LRS (T-LRS) was performed using Adaptive Sampling on an Oxford Nanopore Technologies (ONT) PromethION 24, as previously described [31]. Briefly, ∼5 μg of genomic DNA from all individuals was used to make sequencing libraries using the SQK-LSK114 kit (ONT) following the manufacturer’s instructions. The *GBE1* region was targeted (chr3:79450000-83800000) and *COL1A1* and *FMR1* were used as controls (chr17:50100000-50300000 and chrX:147850000-148050000; GRCh38 coordinates). Approximately 900 ng of prepared libraries were loaded onto a R10.4.1 flow cell and run for 24–48 h on a PromethION running MinKNOW software version 24.06.10. After 24 h, the flow cell was stopped and reloaded if additional data were required. FASTQ files were generated from the raw sequencing data using Dorado 0.9.0 (ONT) using the super accurate model with 5mCG and 5hmCG methylation model 5.0.0 (ONT). Reads were aligned to GRCh38 using minimap2 [32], variants were called using Clair3 [33], followed by phasing using LongPhase [34]. The haplotagged bam files were split by haplotype assignment using samtools view (v1.19) [35] with the argument: *-d HP:1* or *-d HP:2*. We then generated a pileup of the reads aligned to chr3: 81493811-81493824 for each haplotype with pysam (v0.22.1). The pileup was used to count the number of times a base was identified at each position and construct a consensus sequence for each haplotype. Bases with only 1 supporting read at a position were excluded. The consensus sequence for the pathogenic indel was also confirmed visually using Integrative Genomics Viewer [36].

### Short-read RNA-seq

rRNA-depleted total RNA with RIN ≥ 8 was used to generate complementary DNA (cDNA) libraries using the TruSeq stranded total RNA kit (Illumina) according to manufacturer’s protocol. The cDNA libraries were multiplexed and sequenced on a NovaSeq 6000 system (Illumina) for 100 bp paired-end sequencing at a targeted read depth of 150 M per sample. RNA and cDNA library quality were assessed using a Bioanalyzer (Agilent). Preprocessing and alignment to the GRCh38 primary reference assembly (Ensembl v111) were performed using the nextflow rnaseq pipeline (v.3.14.0) (https://doi.org/10.5281/zenodo.1400710) from nf-core [37] with default parameters. In summary, fastqs were preprocessed with Trim Galore (v0.6.7) before alignment to the genome with STAR (v2.7.9a) [38]. We used ggsashimi (v1.1.5) [39] to generate sashimi plots from reads mapped to the reverse strand (sense *GBE1* transcript) and with a minimum of 3 reads per junction. We obtained exon–exon junction read counts from GTEx (V10 adult bulk tissue RNA-seq) [40] to analyze *GBE1* transcript structure in a large cohort of unaffected controls. We visualized the *GBE1* transcript structure and junction counts with ggtranscript (v1.0.0) [41].

### Long-read RNA-seq

Libraries for sequencing were prepared using the cDNA-PCR Sequencing Kit (SQK-PCS114, ONT). Approximately 50 ng of prepared library was loaded onto a R10.4.1 flowcell (ONT) and run for 72 h on a PromethION running MinKNOW software version 24.06.10. Basecalling was performed using Dorado 0.8.1 (ONT) using the super accurate model. We used an internal nextflow pipeline to preprocess and align the ONT cDNA reads. Unmapped bams were converted to FASTQ using samtools FASTQ (v1.21). We then filtered reads by a minimum length of 50 bp and split the FASTQ into smaller files using fastp (v0.23.4) [42] before read preprocessing with pychopper (v2.7.10, edblib backend). We used NCBI’s GCA_000001405.15 (GRCh38, no_alt_analysis_set) reference genome to generate the index for minimap2 (v2.28-r1209) [32] with the parameters: *-k14 -I 1000G*. Reads were then aligned to the reference genome with minimap2 using the following arguments: *-x splice -uf -y --secondary=no -L -a*, and a final, aligned bam file for each sample was generated using samtools merge. We subsampled the bam files, reference genome and annotations to chr3 before running IsoQuant (v3.6.1) [43] with the default parameters for ONT data. The transcript models from IsoQuant were visualized using ggtranscript (v1.0.0) [41].

### *In silico* prediction of off-targets

Hybridization-dependent off-targets of an ASO were predicted by searching the human transcriptome (pre-mRNA and mature mRNA transcripts, GENCODE GRCh38 v45) with BLAST (v2.16.0+) [44, 45] and bowtie (v1.3.1) [46]. For BLAST, we used an *E*-value cutoff of 100 and the scoring parameters for blastn *(-evalue 100 -task blastn*), and for bowtie, alignments were limited to those with a maximum of 2 mismatches (*-v 2*). An alignment was considered an off-target by the following criteria: 0–1 mismatch/gap or 16 contiguous matches, sense direction, distance to exon ≤200 nt. The canonical transcript was used to calculate distance to exon where possible. If an alignment was not within the canonical transcript, the nearest possible exon was used to calculate the distance.

### Immunoblotting

Cells grown in six-well plates were washed once with cold 1× DPBS (without Ca^2+^ and Mg^2+^), then collected using a scraper in 175 μl of cold radioimmunoprecipitation assay (RIPA) buffer (Thermo Fisher, #89900) supplemented with 1× Halt protease inhibitor cocktail (Thermo Fisher, #1861281) and 1× EDTA (Thermo Fisher, #1861274). Samples were incubated on ice for 30 min, then cleared by centrifugation at 15000 × *g* for 10 min at 4°C. Protein concentration in the supernatant was determined using a BCA assay (Thermo Fisher, #A55864). Equal amounts of sample (6–10 μg protein per sample) were mixed with 4× Laemmli buffer (Bio-Rad, #1610747) freshly supplemented with 10% 2-mercaptoethanol (Sigma, #M3148), boiled at 95°C for 5 min, and separated on 4%–20% gradient Criterion TGX stain-free precast gels (Bio-Rad, #5678094) at 130 V for 1.5–2 h. Proteins were transferred to a 0.2 μm polyvinylidene difluoride membrane (Bio-Rad, #162-0175) at 100 V for 30 min, followed by incubation in blocking buffer comprising 1× Tris-buffered saline (TBS) (Bio-Rad, #1706435) with 0.1% Tween 20 (Bio-Rad, #1706531) and 5% blotting grade blocker (Bio-Rad, #1706404) for 1 h at room temperature. Membranes were then incubated overnight at 4°C with the following primary antibodies diluted in blocking buffer: anti-GBE1 (Proteintech, #20313-1-AP, 1:1000) and anti-GAPDH (Santa Cruz, #sc-365062, 1:5000). Membranes were washed four times in TBST (1× TBS with 0.1% Tween 20) for 10 min each, then incubated with appropriate horseradish peroxidase-conjugated (HRP) secondary antibodies (Anti-Rabbit HRP, Bio-Rad, #1706515; Anti-Mouse HRP, Bio-Rad, #1706516) diluted in blocking buffer (1:5000) for 1 h at room temperature. Following another four washes with TBST, the immunoblots were developed using ECL Prime western blotting detection reagent (Cytiva, #RPN2236) and imaged using the Bio-Rad ChemiDoc MP Imaging System with ImageLab software (v3.0.1.14). Densitometry analysis was performed using ImageJ (v2.14.0), and all protein bands were normalized to GAPDH.

### Bjab assay

The BJAB assay was performed as previously described [47]. Briefly, the 1 mM stock ASOs were diluted to 3.2 μM in Bjab assay medium (RPMI + 10% heat inactivated FBS + 1% penicillin-streptomycin) and 50 μl of this dilution was added to 96-well V-bottom plates (Thermo Scientific, #249935). Then, 50 μl of Bjab cells at a density of 50000 cells/well in Bjab assay medium were added to the 96-well plates containing the ASOs. The plates were incubated at 37°C for 24 h to allow for gymnosis. To harvest the cells, the 96-well plate was centrifuged at 1000 x *g* for 5 min and was inverted onto a Kimwipe to remove the supernatant. Forty microliters of GTC lysis buffer was added to each well and RNA was isolated as described earlier. qRT-PCR was performed for *CCL22* using the following primers and probe.

Forward primer: 5′-CGC GTG GTG AAA CAC TTC TA-3′

Reverse primer: 5′-GAT CGG CAC AGA TCT CCT TAT C-3′

Probe: 5′-TGG CGT GGT GTT GCT AAC CTT CA-3′ with 3′ Iowa black, 5′ 6-FAM and internal ZEN modifications

The Bjab assay is performed using a lower concentration of ASOs (1.6 μM) compared to the doses used in other experiments (up to 40 μM). Previous optimization experiments [47] established that this lower concentration is ideal, as it shows sufficient immunogenicity without causing overt cytotoxicity.

### GBE1 enzyme activity assay

GBE activity was assayed as previously described [8]. Briefly, frozen cell pellets were homogenized by oscillator in 100 μl of 5 mM Tris, 1 mM EDTA, 5 mM mercaptoethanol, pH 7.2, and centrifuged at 9200 × *g* for 10 min. Branching enzyme activity was measured by an indirect assay based on incorporation of radioactive glucose-1-phosphate (PerkinElmer Life and Analytical Sciences, Boston, MA) into glycogen by the reverse activity of phosphorylase a (Sigma, St. Louis, MO) as an auxiliary enzyme in a reaction buffer containing 55 mM glucose-1-phosphate, 2 mM AMP, 20 mM MES, pH 6.3, 14 μl/ml 14C glucose-1-phosphate, and 0.3 mg/ml phosphorylase a. At 30-, 45-, and 60-min time intervals, 5 μl of reaction mix was spotted on Whatman^®^ Number 5 qualitative filter paper (Maidstone, England). Unincorporated glucose-1-phosphate was washed for 15 min using 3 changes of 66% v/v ethanol. Glycogen bound radioactive glucose-1-phosphate was quantified by a liquid scintillation counter (Packard Instruments, Boston, MA).

### Statistical analysis

Statistical analysis was conducted using GraphPad Prism software (v10.4.0). All data are presented as the arithmetic mean ± standard deviation (SD). One-way ANOVA followed by post hoc tests was used to compare means across multiple samples, and linear regression with normalized response and variable slope was utilized to calculate IC50 and EC50 values. The specific statistical methods used for each experiment are noted in the figure legends. *P*-value of <.05 was considered statistically significant for all analyses.

## Results

### Characterization of the *GBE1* MT variant

**Figure 1.**
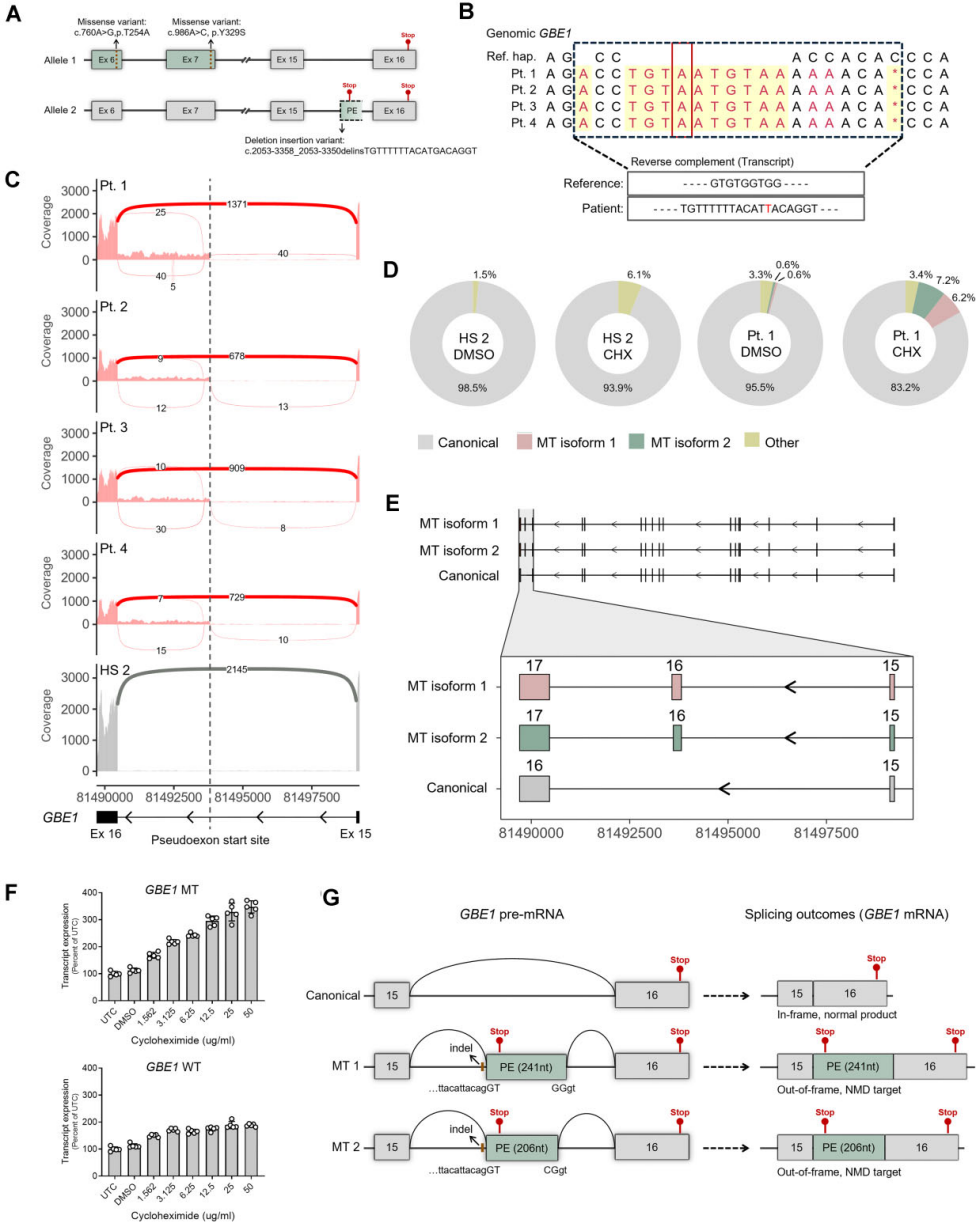
Characterization of the *GBE1* MT variant. (**A**) Schematic depicting the missense and intronic indel mutations present in the *GBE1* gene in our cohort of four APBD patients. Canonical exons are shown in boxes with solid line and the pseudoexon resulting from the usage of the ectopic splice site in the MT transcript is indicated by a box with discontinuous line. Ex = exon; PE = pseudoexon. (**B**) Reference haplotype (Ref. hap.) and the indel consensus sequence identified in each patient from phased WGS reads covering chr3:81493811-81493824. Bases with only 1 read were removed. Non-reference bases are in red, and indels are highlighted in yellow. Deletion is denoted by *. Reverse complement of the indel sequence is given below. (**C**) Sashimi plots comparing short-read RNA-seq reads from fibroblast samples from the four APBD patients (Pt. 1, Pt. 2, Pt. 3, Pt. 4) and a HS control (HS 2) at the *GBE1* exon 15–16 locus (chr3:81489703-81499227). Arcs represent exon junctions with a minimum read count of 3. (**D**) Donut plots showing *GBE1* isoform count proportions per sample. Two noncanonical isoforms have been grouped as “other.” (**E**) Transcript structure of *GBE1* MT isoforms and the canonical isoform identified with long-read RNA-seq. Zoomed inset shows the pseudoexon with exon numbers for each transcript labeled above. (**F**) *GBE1* WT and MT transcript expression in fibroblasts from Pt. 1 treated with varying concentrations of cycloheximide and vehicle control (DMSO) for 16 h. Expression levels were normalized to *PPIA* and are presented as percentage relative to mock treatment. Data represented as mean ± SD. (**G**) Schematic illustrating exon and pseudoexon boundaries at the mutation locus for canonical, MT1 and MT2 pre-mRNAs, and their splicing outcomes at the mRNA level.

We currently have four APBD patients enrolled at n-Lorem, each with a compound heterozygous (in *trans*) mutation in the *GBE1* gene (summarized in Table 1). To the best of our knowledge, these patients are unrelated. Each patient harbors a missense variant (either *GBE1* c.760A > G, p.T254A in exon 6 or *GBE1* c.986A > C, p.Y329S in exon 7) in one allele and shares the same pathogenic intronic indel variant (NM_000158.4(GBE1):c.2053-3358_2053-3350delinsTGTTTTTTACATGACAGGT in intron 15) in the other allele (Fig. 1A). As the primary focus of this study, any further reference to MT variant in this report refers to the indel variant, and not the missense variants. Careful inspection of the patient’s genetic report revealed that Pt. 2 exhibited a single nucleotide polymorphism (SNP) in the indel sequence (c.2053-3358_2053-3350 delinsTGTTTTTTACAT**T**ACAGGT) compared to the published sequence [18], Pt. 1 and Pt. 4 (c.2053-3358_2053-3350 delinsTGTTTTTTACAT**G**ACAGGT) (Table 1). The indel sequence for Pt. 3 was not specified in the genetic report. Interestingly, the diagnostic sequencing method utilized to genotype the patients were Sanger method for Pt. 1 and Pt. 4, and next-generation short-read whole genome sequencing (WGS) for Pt. 2 (Table 1). Since this polymorphism is a few bases upstream of the ectopic splice acceptor site (c.2053-3358_2053-3350 delinsTGTTTTTTACAT[G/T]AC**AG**GT), it is not expected to interfere with the splicing pattern of the MT variant. Therefore, the consequence of the mutation (inclusion of the pseudoexon after canonical exon 15) will remain the same. However, understanding the exact indel sequence is crucial for the design of specific and potent ASOs. Additionally, the complete characterization of the pseudoexon bearing MT transcript is currently lacking, which is essential for the rational design of ASOs as well as for the design of optimal readouts for ASO screens.

**Table 1.** Summary of the indel and missense variants identified in the four GBE1 patients, the sequencing methods used for their diagnosis, and the clinical phenotypes observed

Patient	Indel variant	Missense variant	Diagnostic sequencing method	Clinical phenotypes
Pt. 1	c.2053-3358_2053-3350 delinsTGTTTTTTACATGACAGGT	c.986A > C, p.Y329S	Sanger sequencing	Sensorimotor peripheral neuropathy, spasticity, mild cognitive impairment, neurogenic urinary symptoms
Pt. 2	c.2053-3358_2053-3350 delinsTGTTTTTTACATTACAGGT	c.986A > C, p.Y329S	NGS	Sensorimotor peripheral neuropathy, leg spasticity, optic neuropathy, neurogenic urinary symptoms
Pt. 3	c.2053-3358_2053-3350 delins (sequence not specified)	c.760A > G, p.T254A	Sanger sequencing	Urinary and fecal incontinence, gait dysfunction, loss of proprioception, neurogenic leg weakness
Pt. 4	c.2053-3358_2053-3350 delinsTGTTTTTTACATGACAGGT	c.986A > C, p.Y329S	Sanger sequencing	Urinary incontinence, loss of vibration and proprioception in the lower legs, leg weakness, spasticity, stiff gait

To resolve the discrepancy in the indel sequence, long-read WGS was performed on DNA isolated from blood from all four patients (Pt. 1–4). Analysis of the WGS data showed that the 9-base nucleotide sequence CCACCACAC (reverse complement: GTGTGGTGG) between chr3:81493813-81493821 in the reference haplotype is replaced by the 19-base consensus sequence ACCTGT**A**ATGTAAAAAACA (reverse complement: TGTTTTTTACAT**T**ACAGGT) in all four patient samples (Fig. 1B). Reads with the reference and alternate sequence confirmed that all four patients are heterozygous for this mutation (Supplementary Fig. S1A). To understand the splice changes induced by the indel, short-read total RNA-seq was performed on RNA from fibroblasts derived from all four patient samples and a healthy subject (HS) control. Results confirmed the inclusion of the pseudoexon between exons 15 and 16 in all four patient samples (Pt. 1–4), while it was entirely absent in the HS control (HS 2) (Fig. 1C). Additionally, bulk-tissue RNA-seq data from GTEx [40] were analyzed to examine the splicing of exons 15 and 16 in a large cohort of controls. No evidence of alternative splice junctions was found, further supporting that the pseudoexon inclusion is an aberrant splicing event specifically associated with the indel (Supplementary Fig. S1B). Assessment of exon junction spanning reads at the 3′ end of the pseudoexon, however, suggested the presence of two short MT transcripts resulting from the usage of different splice donor sites and a potential long MT transcript with continuous intron 15 retention extending to exon 16 in all four patient samples (Fig. 1C). Because this analysis was performed on total RNA instead of mRNA-enriched samples, the reads may constitute a mixture of pre-mRNA and mRNA. Combined with the challenges of short-read RNA-seq to unambiguously assemble whole transcripts, it was not possible to decipher the true nature of the MT transcript(s) from this data. Intriguingly, the pseudoexon percent spliced in (PSI) calculated in patient samples was on average 1.7% of the total *GBE1* transcripts (Pt. 1 = 2.8%, Pt. 2 = 1.8%, Pt. 3 = 0.87%, Pt. 4 = 1.3%). Such low pseudoexon PSI could indicate: (i) a low level of ectopic splice site usage or (ii) that the MT transcript is degraded by NMD. Based on previously published data [18], the pseudoexon is the last exon of the MT transcript, and hence, is unlikely to be targeted by NMD. To further understand the lower-than-expected levels of the MT transcript in patient samples and to fully characterize the nature of the MT variant(s) resulting from the indel, long-read mRNA-seq was performed in fibroblasts from Pt. 1 and a HS control (HS 2) treated with DMSO (vehicle) or the NMD inhibitor cycloheximide (50 ug/ml, 16-hr treatment). Analysis using IsoQuant [43] revealed five *GBE1* transcripts across samples—the canonical and two noncanonical transcripts (grouped together and labeled “other”) present in all samples, and two MT isoforms present exclusively in patient samples (Fig. 1D). The two identified MT isoforms are (i) MT isoform 1 with a longer pseudoexon (241 nt) and (ii) MT isoform 2 with a shorter pseudoexon (206 nt) (Fig. 1E). Interestingly, contrary to previous published analysis [18], and consistent with our short-read RNA-seq data (Fig. 1C), both MT isoforms include the complete last canonical exon 16 (Fig. 1E). The two additional noncanonical transcripts (“other”) are shorter transcripts and are not mutation-specific since they are present in both the HS control and the patient samples (Fig. 1D and Supplementary Fig. S1B). We did not identify a complete MT transcript with a larger retained intron 15, as observed in the short-read RNA-seq data. Therefore, this was likely an artifact or unspliced RNA. Quantification of the proportions of the various *GBE1* isoforms in patient cells showed that both MT isoforms 1 and 2 are expressed at roughly the same level in both DMSO and cycloheximide-treated samples (DMSO: MT isoform 1 – 0.6%, MT isoform 2 – 0.6%; Cycloheximide: MT isoform 1 – 6.2%, MT isoform 2 – 7.2%) indicating that both cryptic splice donor sites at the 3′ end of the pseudoexon are used to the same extent, with no preference for one over the other (Fig. 1D). Furthermore, treatment of patient cells with cycloheximide led to a marked increase in both MT isoforms 1 and 2 in comparison to DMSO-treated cells, suggesting that both MT transcripts undergo NMD (Fig. 1D).

A PTC, UAA, is located at the beginning (12 nucleotides from the 5′ end) of the pseudoexon. Given that the pseudoexon is the penultimate exon in both MT isoforms 1 and 2, it is likely to be degraded by NMD, as indicated by the long-read RNA-seq data. To confirm this hypothesis, further experiments utilizing cycloheximide treatment were conducted in patient cells, and the expression levels of *GBE1* MT and canonical/wild-type (WT) transcripts were assessed by qRT-PCR. For the qRT-PCR experiments, two primer probe assays were designed: one targeting the *GBE1* canonical transcript (*GBE1* WT) and the other targeting both MT isoforms (*GBE1* MT) (Supplementary Fig. S2A). Both assays utilize the same forward primer and probe, which target the end of canonical exon 15. The reverse primer for the *GBE1* MT assay was designed to the beginning of the pseudoexon, a region shared by both MT isoforms 1 and 2, allowing for the identification of both MT transcripts. The reverse primer for *GBE1* WT assay was designed to the 5′ region of canonical exon 16. Since both the normal and the MT *GBE1* isoforms include canonical exon 16, *GBE1* WT assay is not specific and will recognize all three transcripts (canonical, MT isoform 1, and MT isoform 2). Although designing a reverse primer that spans exon 15–16 junction would have been ideal, the high GC content and presence of mononucleotide repeat sequence in this region hindered the successful design of such primers. According to RNA-seq data, MT isoforms constitute a small percentage of the total *GBE1* transcripts in patient cells at baseline (short-read RNA-seq: 1.7%; long-read RNA-seq: 1.2%). Therefore, the majority of transcripts identified by the *GBE1* WT assay in qRT-PCR experiments will originate from the canonical *GBE1* transcript, with minimal interference from the MT isoforms. To validate the *GBE1* WT and MT primer-probe assays, transcript expression analyses were performed using RNA from control and patient samples. As anticipated, qRT-PCR with the *GBE1* WT assay demonstrated that the canonical *GBE1* transcript was reduced in all four patient samples (Pt. 1–4) compared to two HS controls (HS 1 and HS 2) (Supplementary Fig. S2B). Furthermore, treatment of HeLa and a HS fibroblast cell line with 30 nM of siRNA targeting *GBE1* (siGBE1) for 3 days resulted in a significant reduction of the *GBE1* WT transcript (96% reduction in HeLa and 93.6% reduction in fibroblasts) compared to cells treated with a scrambled control (siSCR), further confirming the specificity of the assay (Supplementary Fig. S2C). The qRT-PCR conducted with the *GBE1* MT assay showed amplification of the target exclusively in the four patient samples (Pt. 1–4), with no amplification detected in the control samples (HS 1, HS 2), thereby confirming the specificity of the readout (Supplementary Fig. S2D).

To confirm that MT isoforms 1 and 2 are degraded by NMD, RNA was isolated from fibroblasts of Pt. 1 treated with varying concentrations of cycloheximide (a 6-point, two-fold serial dilution starting at 50 ug/ml) for 7 and 16 h, and qRT-PCR was performed using *GBE1* WT and MT assays. The data demonstrate a clear dose-dependent increase in *GBE1* MT transcript following cycloheximide treatment at both 7-hr (Supplementary Fig. S1D) and 16-hr (Fig. 1F) time points, confirming that the *GBE1* MT products, contrary to previously published literature, are indeed targets of NMD. A slight dose-dependent increase was also observed in the *GBE1* WT transcript at both time points (Supplementary Fig. S1D and Fig. 1F). This increase may be attributed to: (i) an overall elevation of all transcripts due to the inhibition of translation by cycloheximide and/or (ii) enhanced detection of *GBE1* MT transcripts by the *GBE1* WT primer-probe assay. Altogether, these findings demonstrate that the pathogenic *GBE1* intronic indel mutation (NM_000158.4(GBE1):c.2053-3358_2053-3350delinsTGTTTTTTACATTACAGGT) alters splicing, resulting in the production of two MT isoforms that include a pseudoexon between canonical exons 15 and 16. The inclusion of this pseudoexon in both MT isoforms generates out-of-frame products with a PTC, which are subsequently degraded by cellular NMD (Fig. 1G).

### High-throughput screening identifies multiple ASOs that modulate *GBE1* transcripts in a concentration-dependent manner

**Figure 2.**
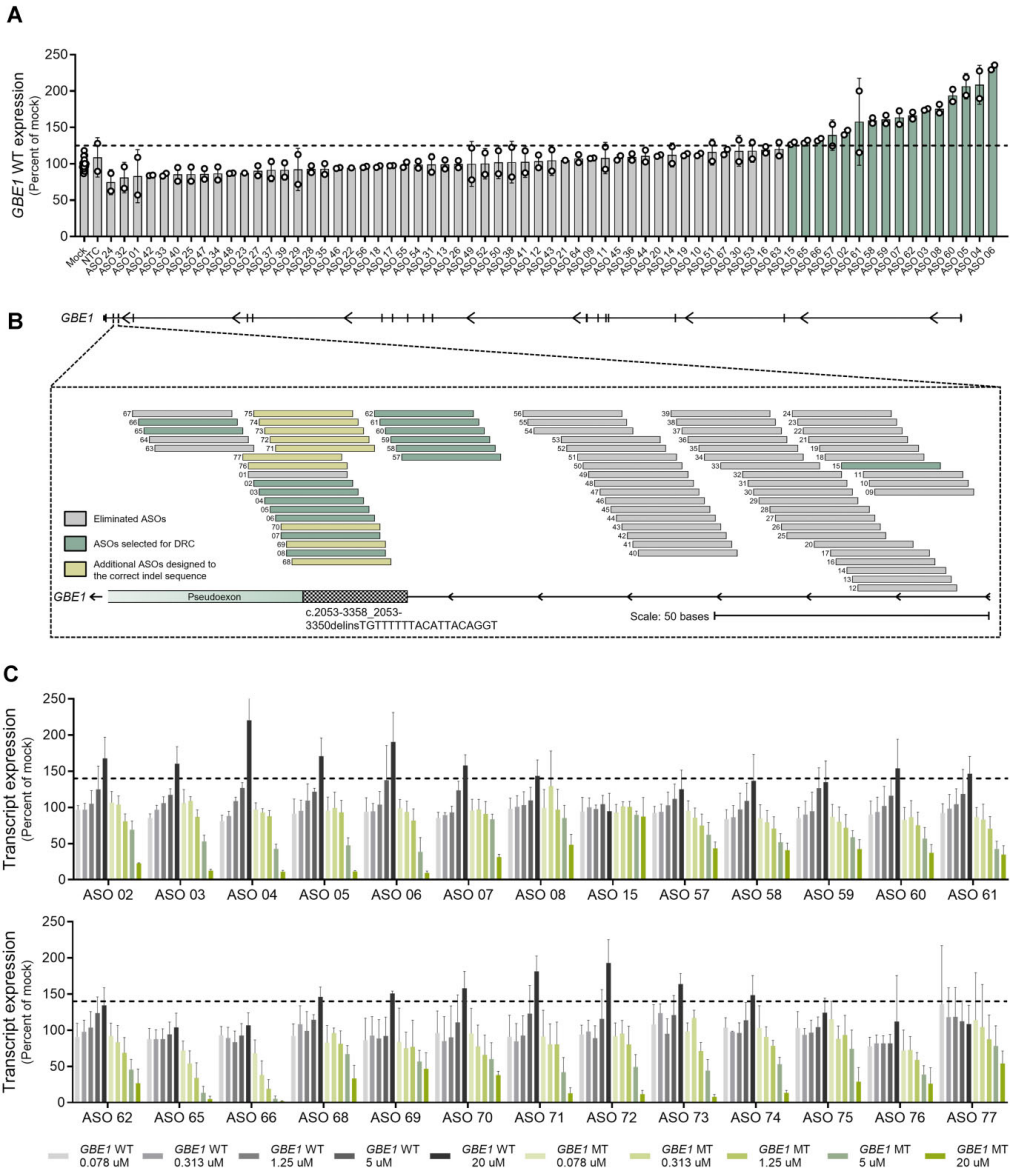
Single-dose and dose-response screenings identify lead ASOs that modulate *GBE1* transcripts in a concentration-dependent manner. (**A**) *GBE1* WT transcript expression in fibroblasts from Pt. 1 treated with 67 ASOs (ASOs 1–67) at 20 μM for 16–20 h. A NTC control ASO was included as negative control. Data points represent technical replicates (2 per ASO). Expression levels were normalized to *PPIA* and are presented as percentage relative to mock treatment. Green bars represent the 16 ASOs that upregulated *GBE1* WT transcript by 125% or 1.25-fold of mock (dashed line). (**B**) Schematic depicting alignment of all tested ASOs to the *GBE1* transcript. The diagram shows ASOs that were eliminated during the single-dose screen, ASOs that were selected to proceed to DRC and the additional 10 ASOs (ASOs 68–77) that were designed to the correct indel sequence. (**C**) *GBE1* WT and MT transcript expression measured by qRT-PCR in fibroblast cells from Pt. 1 treated with varying concentrations of ASOs (five-point, four-fold serial dilution starting at 20 μM) for 18–24 h (technical replicates = 4–12 per ASO/condition). Expression levels were normalized to *PPIA* and are presented as percentage relative to mock treatment. The dashed line represents a threshold of 140% or 1.4-fold of mock. All data are represented as mean ± SD.

Pathogenic deep intronic mutations that result in the utilization of ectopic splice sites and subsequent inclusion of pseudoexons, such as the one described in this study, are ideal targets for splice-modulating ASOs. To block the usage of the ectopic splice site and redirect splicing to the canonical pattern, we designed 18-mer RNA oligonucleotides uniformly modified with phosphorothioate backbone (PS) and methoxyethyl at 2′ position of the ribose moiety (2′MOE). The region surrounding the indel was tiled at 1-nucleotide resolution. ASOs with GC content outside the 25%–75% range, homopolymer repeats of six or more, and significant off-target hybridization were filtered out, resulting in 67 ASOs for screening (Supplementary Table S1). This initial set of ASOs was designed prior to the detailed characterization of the MT variant, and hence, was designed to the reported indel sequence (reported indel sequence: TGTTTTTTACAT**G**ACAGGT; correct indel sequence: TGTTTTTTACAT**T**ACAGGT). To identify the ASOs that modulate splicing of the pseudoexon, single-dose screening was conducted using all 67 ASOs in fibroblast cells derived from Pt.1. Cells were electroporated with 20 μM ASO, and transcript changes were assessed 18–24 h later by qRT-PCR using the *GBE1* WT assay. A length- and chemistry-matched nontargeting control (NTC) ASO, with no perfect complementarity to any transcript in the human genome was used as a negative control [48]. qRT-PCR identified 16 ASOs that increased *GBE1* WT transcript by >125% (1.25-fold relative to the mock), with NTC-treated cells showing results similar to the mock control (Fig. 2A). The *GBE1* WT assay amplifies a region spanning exons 15 and 16, capturing both the target allele (which contains the indel variant) and the nontarget allele (which has the missense variant). Therefore, any qRT-PCR quantification for the *GBE1* WT transcript obtained with this assay will reflect the *GBE1* WT transcript from both alleles. Hence, although a low threshold of 125% was applied in this analysis, the actual increase in *GBE1* WT transcript derived solely from the target allele is expected to be significantly greater. Aligning all ASOs along the *GBE1* transcript revealed that all effective ASOs (green bars), except for three (ASOs 15, 65, and 66), bind directly to the indel (Fig. 2B). Notably, the three ASOs targeting regions outside the indel (ASOs 15, 65, and 66) were the least effective in upregulating the *GBE1* WT transcript (Fig. 2A). Following the identification of the correct indel sequence, an additional 10 ASOs (yellow bars) that perfectly match this sequence were designed and advanced for further studies (Fig. 2B and Supplementary Table S1).

To further refine the selection of the most effective ASOs, a dose-response study was performed using all 26 ASOs (16 from single-dose screening plus the additional 10 designed to the correct indel sequence). Fibroblasts from Pt.1 were electroporated with varying concentrations of ASOs (five-point, four-fold serial dilution at 0.078–20 μM) and transcript changes were assessed 18–24 h later by qRT-PCR using both *GBE1* WT and MT assays. Out of the 26 tested ASOs, 21 (ASOs 2–8, 57–62, 68–75) demonstrated clear concentration-dependent upregulation of the *GBE1* WT transcript, accompanied by simultaneous downregulation of the *GBE1* MT transcript (Fig. 2C). Among the 21 ASOs that showed a positive response, 16 ASOs (ASOs 2–8, 60–61, 68–74) that increased *GBE1* WT transcript levels by >140% (1.4-fold relative to the mock) at the highest concentration (20 μM) were identified as the most effective and were selected as lead ASOs for further functional validation. Of particular interest, the top 16 ASOs included both perfectly matched and mismatched sequences to the indel, indicating that single nucleotide mismatches are well tolerated, as expected. ASO 15, which had shown a positive response in the initial screening, dropped out in the dose-response study. This length- and chemistry-matched ASO, designed to a region close to the indel, served as the negative control for all subsequent experiments. Dose-response curves (DRCs) for the lead ASOs, derived from the data in Fig. 2C, along with calculations of the absolute half-maximal effective concentration (EC_50_) and inhibitory concentration (IC_50_), are presented in Supplementary Fig. S3A. The absolute EC_50_ and IC_50_ for each ASO were calculated by fitting the data using a normalized response and variable slope model. For absolute EC_50_ quantification, the *GBE1* WT transcript values were adjusted by subtracting a baseline value of 100 (equivalent to the percentage of mock treatment) from all values. This adjusted value, referred to as “*GBE1* WT (% transcript – 100),” was used to generate the DRCs and calculate the absolute EC_50_ values. Due to biological variability, some *GBE1* WT transcript levels from qRT-PCR data at lower concentrations end up lower than the average of the control (mock treatment) (e.g. ASO 03 in Fig. 2C), and hence appear as negative when adjusted.

ASOs with phosphorothioate backbone can activate the cellular innate immune pathway through toll-like receptor 9 [49, 50]. To assess the immune activation potential of these ASOs and to exclude those with proinflammatory effects from further development, a recently developed high-throughput *in vitro* assay was employed [47]. The capacity of ASOs to induce innate immune activation was evaluated by treatment of Bjab cells with all 26 test ASOs (used for dose-response analysis) at a concentration of 1.6 μM for 24 h followed by measurement of *CCL22* transcript levels. All 26 tested ASOs were confirmed to be safe, as none resulted in increased *CCL22* levels (Supplementary Fig. S4A). Known ASOs (ASO 10438, ASO 735746, ASO 785674, ASO 353512), previously identified as non-/pro-inflammatory in clinical trials, were included as benchmark controls in the assay. Collectively, these data demonstrate that screening in APBD patient cells has identified several lead ASOs that effectively block the pseudoexon and enhance WT transcript levels in a dose-dependent manner. Importantly, all identified lead ASOs were confirmed to be safe and did not elicit an inflammatory response *in vitro*.

### Lead ASOs increase GBE1 protein levels and enzyme activity in APBD patient cells

**Figure 3.**
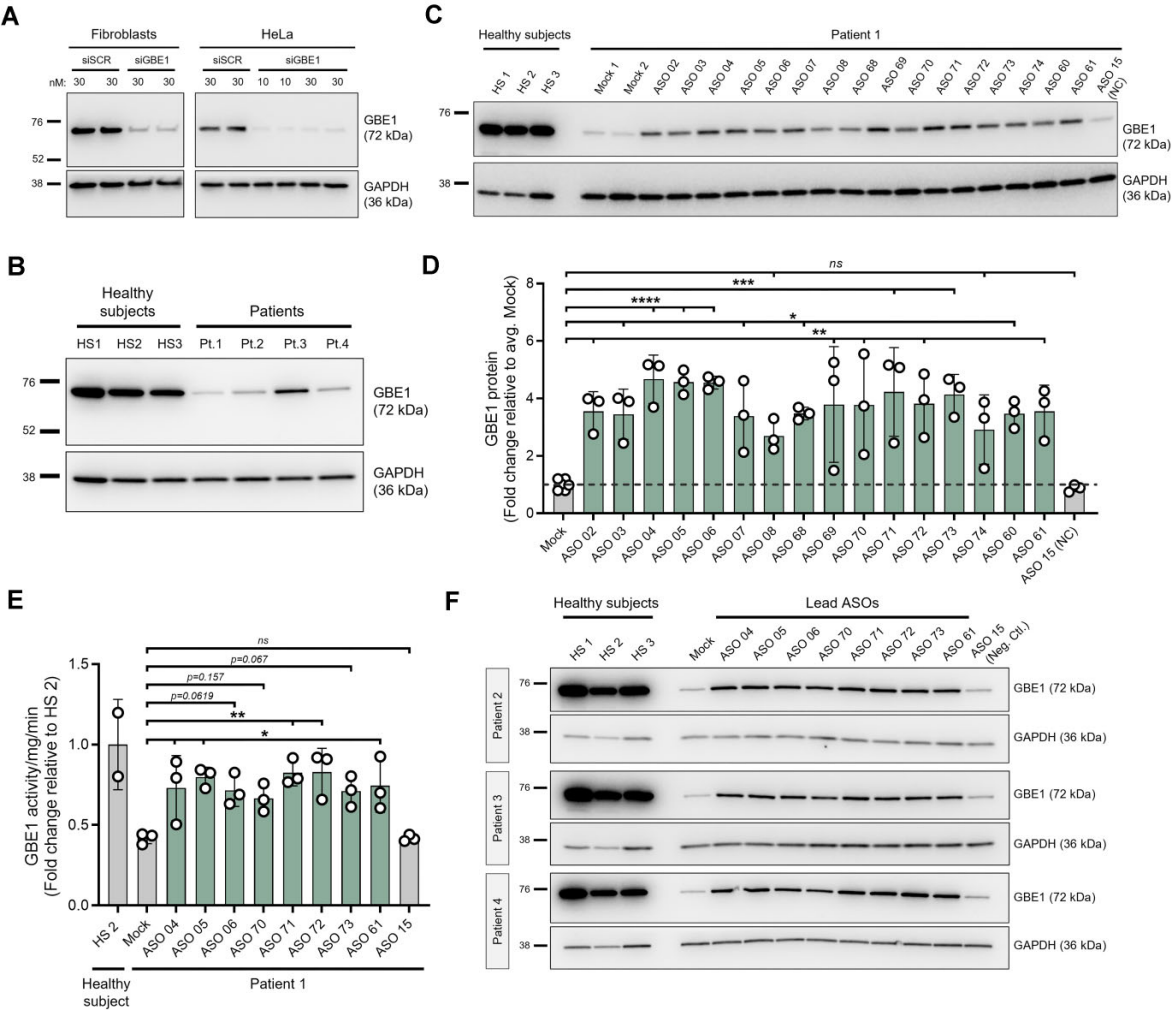
ASO-mediated rescue of GBE1 protein and enzyme activity in patient cells. (**A**) Immunoblot performed on HS control fibroblast and HeLa cells treated with varying concentrations of scrambled (siSCR) or *GBE1*-targeting siRNA (siGBE1) for 3 days. (**B**) Immunoblot comparing baseline GBE1 expression between three HS control fibroblasts (HS1, HS2, HS3) and the four APBD patients (Pt.1, Pt.2, Pt.3, Pt.4). (**C**) Representative image and (**D**) quantitation of GBE1 protein levels in fibroblasts from Pt. 1 treated with the 16 lead ASOs at 40 μM for 4 days (*n* = 3 independent biological replicates). ASO 15 was included as a negative control (NC) and three HS fibroblast samples (HS 1, HS 2, HS 3) were included as positive controls. The dashed line represents fold change equal to 1. Statistical analysis comparing the means of all treatment conditions to the mean of Mock was performed using one-way ANOVA followed by Dunnett’s post hoc analysis. ns, *p* < .05; *p < .05; **p < .01; ***p< .001; ****p< .0001. (**E**) GBE1 enzyme activity measurement in patient fibroblast cells treated with lead ASOs at 40 μM for 4 days. ASO 15 was included as a negative control and a HS fibroblast sample (HS 2) was used as the positive control. The dashed line denotes fold change equal to 1. Data points represent independent biological replicates (*n* = 3). Statistical analysis comparing the means of all treatment conditions to the mean of Mock was performed using one-way ANOVA followed by Dunnett’s post hoc analysis. ns, *p*< .05; *p< .05, **p < .01. (**F**) Immunoblot was performed in Pt. 2, 3, and 4 fibroblasts treated with eight lead ASOs at 40 μM for 4 days. All data presented as mean ± SD. GAPDH was used as the loading control for all immunoblots.

To investigate the effects of lead ASOs on GBE1 protein expression and functionality, additional biochemical and functional validations were performed. A polyclonal GBE1 antibody, generated against the first 302 amino acids of the protein, was employed to quantify overall cellular GBE1 protein levels. Immunoblotting of HeLa cells and fibroblasts from healthy subjects treated with varying concentrations of siRNA (10 or 30 nM for 72 h) targeting *GBE1* revealed a significant reduction of GBE1 protein compared to the scramble control (siSCR), thereby confirming the specificity of the antibody (Fig. 3A). Consistent with previously published findings [18], baseline protein expression analysis indicated that all four patients (Pt. 1–4) exhibited reduced GBE1 protein levels relative to three healthy subject fibroblast lines (HS 1–3) (Fig. 3B). To evaluate the impact of ASOs on the restoration of GBE1 protein levels, fibroblasts from Pt. 1 were electroporated with all 16 lead ASOs at a concentration of 40 μM, and samples were collected four days post-treatment. Immunoblotting and quantification showed that 14 (ASOs 02–07, 68–73, 60–61) out of the tested 16 ASOs led to significant increase in GBE1 protein levels by at least 3.4-fold compared to mock treatment, while ASO 15, included as a negative control, exhibited no effect. The last two ASOs, even though not significant, showed a trend for upregulation (ASO 08, *p*= .187; ASO 74, *p*= .092) (Fig. 3C and D, and Supplementary Table S2). To assess the functionality of the upregulated protein, GBE1 enzymatic activity was performed in samples from Pt. 1 fibroblasts electroporated with 40 μM of the eight most effective lead ASOs (selected based on immunoblot data; ASO 69 was excluded from this list due to high variability in the data) for 4 days. Relative quantification showed that the basal GBE1 enzyme activity in patient cells (mock treatment) was 0.41-fold of healthy subject control. Treatment with 5 (ASOs 04, 05, 71, 72, 61) out of the eight lead ASOs led to significant increase in GBE1 enzyme activity levels compared to mock-treated controls. The rest 3 ASOs showed a trend toward increased GBE1 enzyme activity levels (ASO 06, *p*= .062; ASO 70, *p*= .157; ASO 73, *p*= .067), while ASO 15-treated cells exhibited no change (Fig. 3E and Supplementary Table S2). The increase in GBE1 enzyme activity across all eight ASOs was an average of 0.75-fold (range: 0.67–0.83-fold) of healthy subject control. Further validation was performed by testing the ASOs in three additional patient-derived fibroblast lines. Fibroblasts from patients 2, 3, and 4 were treated with 40 μM of the top eight ASOs, and protein changes were assessed in samples collected 4 days post-treatment. Consistent with previous findings, immunoblotting and quantitation demonstrated that treatment with six out of the eight ASOs resulted in a significant increase in GBE1 protein levels by at least 3.08-fold compared to mock-treated controls in all three patient cell lines. The remaining two ASOs exhibited a trend toward an increase in GBE1 protein levels (ASO 70, *p*= .29; ASO 61, *p*= .126) (Fig. 3F, Supplementary Fig. S5A, and Supplementary Table S2). Further quantitative analysis revealed a strong positive correlation (*R*^2^= 0.8) between ASO-mediated alteration in GBE1 protein levels and enzyme activity (Supplementary Fig. S5B). Lastly, *in silico* analysis was conducted to identify potential hybridization off-targets, and no transcripts with regions identical to the binding sites of the 16 lead ASOs were identified. The off-target analysis was further extended to exonic and intronic regions within 200 nucleotides of an exon that have a mismatch or 16 contiguous bases identical to a lead ASO’s binding site. While potential off-target effects were identified for four of the lead ASOs (ASOs 61, 69, 72, 73), majority of the lead ASOs (remaining 12 candidates) showed no predicted off-targets (Supplementary Fig. S5C). Taken together, these results provide evidence that ASO-mediated correction of aberrant splicing significantly improves functional GBE1 protein levels in relevant *in vitro* disease models.

## Discussion

WGS combined with RNA-seq is an effective means to characterize deep intronic pathogenic variants and their impact on the transcriptional profile of a gene. An intriguing and unexpected finding from our long-read WGS of four APBD patients revealed that they all share the same pathogenic indel (c.2053-3358_2053-3350 delinsTGTTTTTTACATTACAGGT), which differs from the previously reported indel sequence (c.2053-3358_2053-3350 delinsTGTTTTTTACATGACAGGT) by one nucleotide [18]. This discrepancy suggests two possibilities: (i) the existence of a previously unreported SNP in the indel or (ii) a mischaracterization of the indel in earlier studies. The initial characterization of this indel was performed using the Sanger sequencing method [18]. While Sanger sequencing is a reliable and cost-effective method for identifying SNPs, it is not ideal for resolving complex heterozygous deletion-insertion mutations like the one described in this study. Notably, the two patients (Pt. 1 and Pt. 4) within our cohort who were mis-genotyped during the initial diagnostic evaluation were both sequenced using the Sanger method, whereas Pt. 2 who was correctly genotyped was sequenced using NGS (Table 1). No SNP in the indel was observed in the cohort of sixteen patients from the initial study [18], nor in the cohort of four unrelated APBD patients in this study. Although we cannot conclusively determine the reason for the observed discrepancy in the indel sequence without further experimentation, the evidence mentioned above suggests a higher likelihood of previous mischaracterization of the indel variant. It is important to note here that both indel sequences (delinsTGTTTTTTACAT[G/T]ACAGGT) result in the same aberrant splice change. However, in order to design specific ASOs, it was crucial for us to understand the precise indel sequence in our patients.

Our in-depth investigation employing both short- and long-read RNA-seq has revealed several novel aspects of the MT transcripts. First, the indel mutation produces two distinct MT isoforms, both expressed at comparable levels. These MT transcripts are polyadenylated, as demonstrated by the use of poly-dT primers in the preparation of the long-read RNA-seq libraries. Additionally, both isoforms utilize the same splice acceptor site, consistent with the start of the pseudoexon as identified in prior research [18]. Second, contrary to prior understanding, both MT isoforms include the canonical last exon (exon 16) of the *GBE1* transcript, with the pseudoexon forming the penultimate exon. Lastly, experiments with cycloheximide treatment confirmed that both MT isoforms are targets of cellular NMD due to the PTC present at the 5′ end of the pseudoexon. The small fraction of MT isoforms that escape NMD will result in an out-of-frame, truncated GBE1 protein that is likely dysfunctional. Throughout the study, we addressed several discrepancies related to the mutation. Failure to determine if NMD was likely by treating with cycloheximide, the identification of the pseudoexon as the last exon of the MT transcript, and the use of non-quantitative methods such as Sanger sequencing to characterize aberrant splicing likely led to the misleading findings regarding this particular *GBE1* mutation. Addressing these issues is crucial for the successful discovery and development of therapeutic ASOs and demonstrates the need for in-depth molecular studies and high-resolution, long-read sequencing methods to accurately decipher and validate complex indel mutations. Even though we performed long-read RNA-seq in only one patient, the MT isoforms should be consistent across all four APBD patients, as WGS did not identify any subject-specific variants in the pseudoexon region that could affect splicing, and short-read RNA-seq showed the same pattern of exon junction reads across all four patients.

High-throughput screening in patient-derived cells identified 16 lead ASOs that effectively blocked the ectopic splice site and improved GBE1 protein levels in four different APBD patient cell lines. Further functional studies confirmed that treatment with eight of these lead ASOs significantly improved GBE1 enzyme activity compared to mock-treated controls. Although the remaining eight lead ASOs were not tested in the enzyme activity assay, it is highly likely they would demonstrate a similar effect, as they all tile around the same target region (Fig. 2B). The functional assay used here is semi-quantitative and does not provide an absolute measurement of enzyme activity. Relative quantification across samples showed that treatment with the eight lead ASOs increased GBE1 enzyme activity to an average of 0.75-fold of the healthy control level (Fig. 3E). Restoration of enzyme function to levels observed in healthy subjects is not achievable due to the compound heterozygous (in *trans*) mutations in the *GBE1* gene of these patients. While ASO-mediated correction of the mis-splicing caused by the indel variant in one allele is possible, the presence of a missense variant in the second allele continues to compromise protein functionality. Given the compound heterozygous nature of the mutation, the ideal control to use in biochemical and functional assays would be cells from an unaffected carrier heterozygous for the same missense mutation as the patients. Nonetheless, an ASO-mediated improvement in GBE1 enzyme activity to 0.75-fold, which is more than halfway between the patient sample (0.41-fold) and the healthy control (one-fold), strongly indicates a restoration of enzyme levels to those of an unaffected heterozygous individual. A previous study testing a gene therapy approach for APBD demonstrated that intravenous injection of AAV9-mediated human *GBE1* into 14-day-old APBD mice [8] resulted in a significant increase in GBE1 enzyme activity in the brain, skeletal muscle, and heart at 3 months of age [12]. This increase led to a systemic reduction of accumulated glycogen in the brain, skeletal muscle and liver, and significantly improved muscle strength and coordination, as measured by the Rota-rod test, suggesting a potential correction of central and peripheral nervous system dysfunction. Notably, a 1.5-fold increase in GBE1 enzyme activity was sufficient to significantly reduce glycogen levels in the cerebrum of these APBD mice [12]. This study provides an important proof-of-concept in a relevant *in vivo* disease model that enhancing GBE1 levels can effectively reduce glycogen storage and ameliorate the pathogenic features of the disease, thereby supporting our therapeutic strategy.

Changes in insoluble glycogen levels and PBs would further validate our approach; however, the cell system used in this study does not allow for such assessments. Fibroblasts inherently lack the ability to store glycogen, and as a result, APBD patient-derived fibroblasts do not display measurable phenotypes related to glycogen accumulation. Efforts to induce glycogen accumulation and PB formation in these fibroblasts using cobalt chloride treatment, as described in a previous study, did not yield reliable results (data not shown) [11].

Due to the discrepancy in the indel sequence, which was later resolved by WGS, we designed two sets of ASOs targeting the *GBE1* indel. The first set was based on the previously published sequence (mismatch ASOs), and the second set was designed against the correct indel sequence (perfect match ASOs) (Fig. 2B). Both sets of ASOs were used in screening, biochemical, and functional analyses. Interestingly, all tested mismatch ASOs upregulated GBE1 protein and enzyme activity as effectively as their corresponding perfect match ASOs (ASO 03 – ASO 74; ASO 04 – ASO 73; ASO 05 – ASO 72; ASO 06 – ASO 71; ASO 07 – ASO 70; ASO 08 – ASO 69). This confirms that single nucleotide mismatches within a target region are well tolerated for splice-switching ASOs. Consistent with our findings, a previous study reported similar results with ASOs designed to skip exon 25 of the dystrophin gene [51]. These observations suggest that mismatch ASOs could be a viable therapeutic option, potentially eliminating the need to design sequence-specific ASOs for each SNP to achieve steric blocking.

Ongoing research is exploring several therapeutic approaches for APBD, including small molecules, miRNA, and CRISPR/Cas9 techniques aimed at reducing glycogen synthase 1 to decrease GBE1 substrate levels [9–11], gene therapy to enhance GBE1 enzyme activity [12], and small molecules to promote lysosomal degradation of PBs [13]. One promising approach involves a small molecule that blocks the lysosomal membrane protein LAMP1 to increase autophagic flux, thereby promoting the lysosomal degradation of PBs. This molecule, GHF201, was recently granted orphan drug designation by the U.S. Food and Drug Administration (FDA) (https://www.accessdata.fda.gov/scripts/opdlisting/oopd/detailedIndex.cfm?cfgridkey=953523) and is currently in clinical development [13, 52, 53]. Systemic administration of this small molecule to APBD mice led to significant improvements in survival and motor function, and reduced PBs and glycogen levels in the brain, peripheral tissues such as liver and heart, and peripheral nerves [13]. As a general activator of autophagy, GHF201 can be utilized as a treatment for other glycogen storage diseases and has been shown to alleviate pathology in *in vitro* and *in vivo* models of Type III glycogenosis [54]. However, a recent study reported that GHF201 failed to alter PBs in a mouse model of Lafora disease, suggesting that the effectiveness of GHF201 in clearing PBs may vary between different glycogen storage diseases [52]. This discrepancy, potentially due to differences in the chemical nature of PBs or a primary defect in autophagy in Lafora disease, warrants further investigation [52]. The clinical development of GHF201 for APBD is promising. Unlike the splice-modulating ASOs developed in our study that target one specific common APBD mutation, GHF201 can be used in all APBD cases regardless of the causal mutation. However, a long-term broad upregulation of a key physiological process such as autophagy could have detrimental effects. In contrast, the ASOs developed in this study, which target the root cause of the disease, offer a more precise targeting of the *GBE1* indel, thus providing a better level of specificity.

Over the years, the FDA has approved five splice-modulating ASOs targeting spinal muscular atrophy (SMA) and Duchenne muscular dystrophy [55]. Among these, nusinersen, a 2′MOE/PS-modified ASO designed to treat all types of SMA, has demonstrated excellent safety and efficacy in long-term studies [56]. Compared to other classes of drugs, ASOs are a particularly attractive therapeutic modality for tailored therapies due to several factors: ease of design against specific target sequences, well-characterized safety and efficacy data from clinical trials, consistent behavior within specific chemical classes, well-studied routes of administration, and favorable FDA regulations [57, 58]. The development of ASOs for n-of-1 conditions began with an ASO designed to skip a cryptic splice variant in a child with neuronal ceroid lipofuscinosis 7 [59]. Despite multiple studies reporting the discovery of ASOs for n-of-1 conditions [27, 60, 61], the limited number of treatment-amenable patients makes the development of tailored therapies commercially unviable. To address this, nonprofit organizations like ours (n-Lorem Foundation) and several others have been established with the primary goal of developing tailored ASOs for ultra-rare n-of-1 or n-of-few cases and are expected to have a significant impact [62–64]. In conclusion, our work outlines the discovery of clinically relevant ASOs to correct aberrant splicing in a subset of APBD patients. More broadly, this study highlights how precise targeting of deep intronic variants using ASOs can lead to the discovery and potential development of tailored therapies for various genetic diseases with similar pathogenic mutations, highlighting significant implications for personalized medicine.

## Supplementary Material

gkaf658_Supplemental_Files

## Data Availability

Processed RNA-seq data are available at Zenodo (DOI 10.5281/zenodo.14934140). Raw RNA-seq and genetic data may be provided upon request, subject to an internal review to ensure patient confidentiality.
