# Supplementary material for: Splice-modulating antisense oligonucleotides targeting a pathogenic intronic variant in adult polyglucosan body disease correct mis-splicing and restore enzyme activity in patient cells
Source: Nucleic Acids Res. 2025 Jul 16;53(13):gkaf658. doi: 10.1093/nar/gkaf658 (PMC12266137; doi:10.1093/nar/gkaf658)
Supplement: gkaf658_Supplemental_Files [file gkaf658_supplemental_files.zip › NAR_Supl_material.pdf]

SUPPLEMENTARY DATA

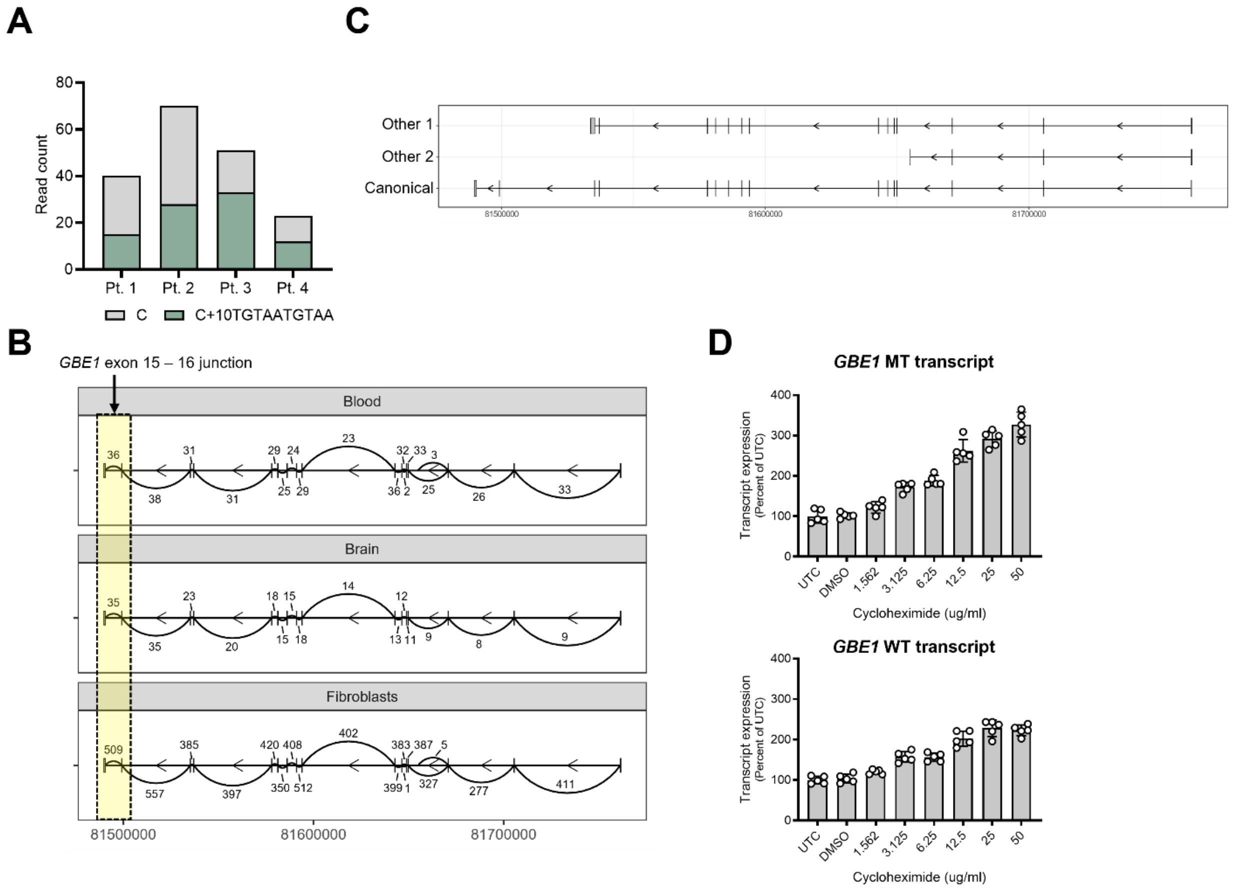

Supplementary Figure 1: Characterization of the mutant *GBE1* variant. (A) Bar graph shows counts of bases from reads at chr3:81493814 from WGS of the four patient samples (Pt. 1, Pt. 2, Pt. 3, Pt. 4). **(B)** *GBE1* average splice junction read counts in blood, brain and fibroblasts from GTEx v10 bulk-tissue RNA-seq data. Splice junctions are depicted by arcs with the canonical *GBE1* transcript structure shown. Reads across the *GBE1* exon 15-16 junction are highlighted in yellow. **(C)** Transcript structure of *GBE1* noncanonical ("other") and canonical isoform identified with long-read RNA-sequencing. **(D)** *GBE1* WT and MT transcript expression in fibroblasts from Pt. 1 treated with varying concentrations of cycloheximide and vehicle control (DMSO) for 7 hours. Expression levels were normalized to *PPIA* and are presented as percentage relative to mock treatment.

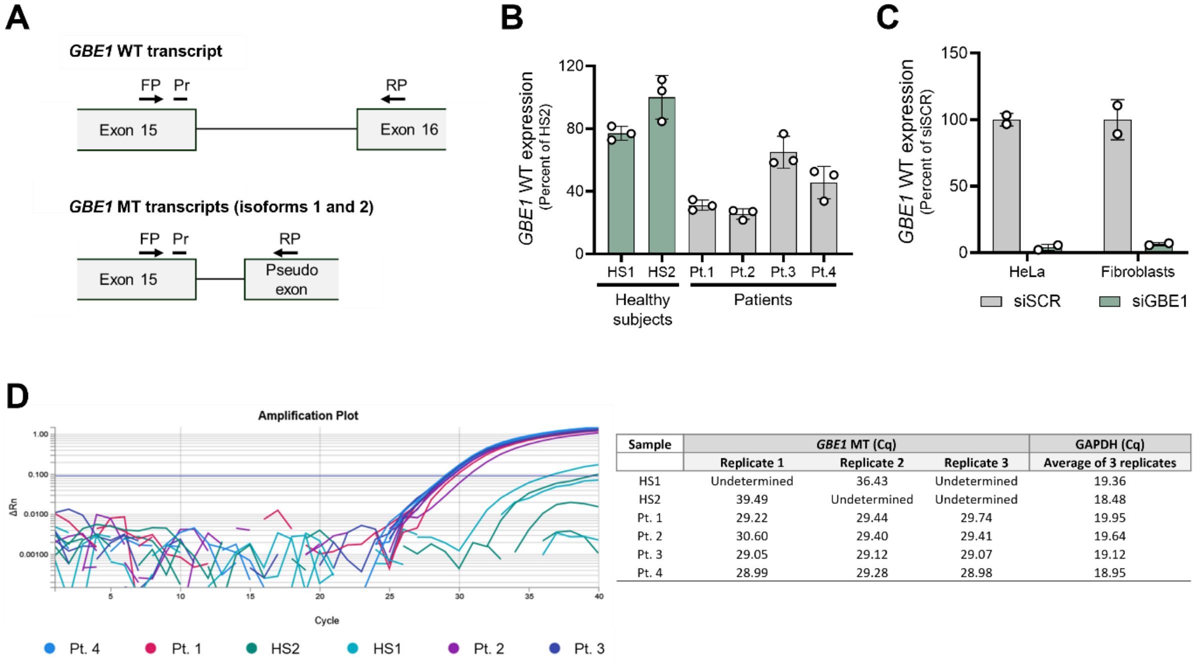

Sample	*GBE1* MT (Cq)			GAPDH (Cq)
	Replicate 1	Replicate 2	Replicate 3	Average of 3 replicates
HS1	Undetermined	36.43	Undetermined	19.36
HS2	39.49	Undetermined	Undetermined	18.48
Pt. 1	29.22	29.44	29.74	19.95
Pt. 2	30.60	29.40	29.41	19.64
Pt. 3	29.05	29.12	29.07	19.12
Pt. 4	28.99	29.28	28.98	18.95

Supplementary Figure 2: Validation of *GBE1* WT and MT primer probes. (A) Schematic depicting location of the forward primers (FP), the reverse primers (RP) and the probes (Pr) designed to detect *GBE1* WT and MT transcripts. **(B)** Comparison of *GBE1* WT transcript expression between HS controls (HS1, HS2) and patient (Pt.1, Pt.2, Pt.3, Pt.4) samples. Expression levels were normalized to *GAPDH* and are presented as percentage relative to HS2. **(C)** *GBE1* WT transcript changes in HeLa and control fibroblast cells treated with scrambled (siSCR) or GBE1 targeting (siGBE1) siRNA at 30nM for 3 days. Expression levels were normalized to *PPIA* and are presented as percentage relative to siSCR. **(D)** Amplification plot and raw Cq values from qRT-PCR for *GBE1* MT transcript across HS (HS1, HS2) and patient samples (Pt. 1, Pt. 2, Pt. 3, Pt. 4). *GADPH* was used as the housekeeping gene to control for input amount.

A

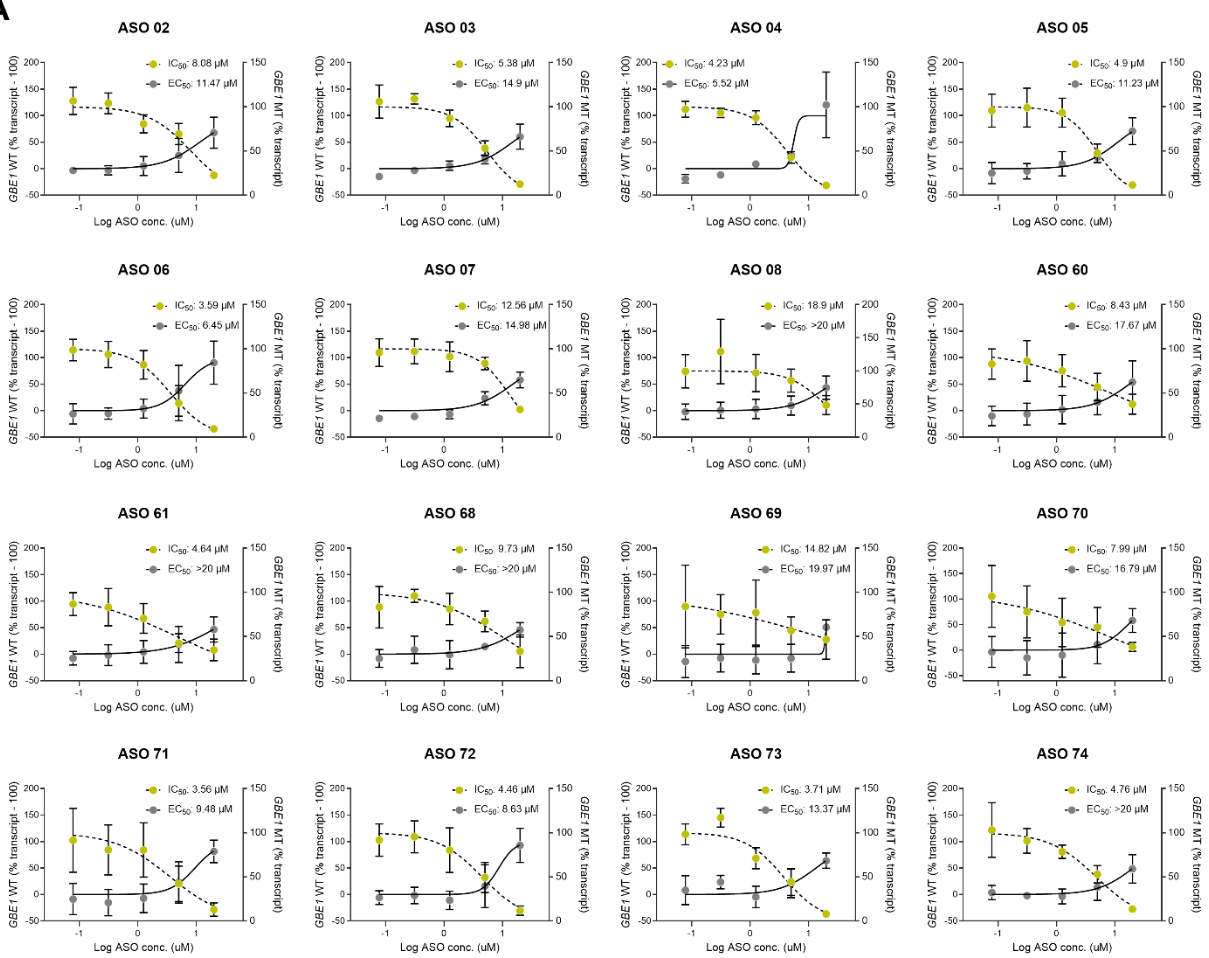

Supplementary Figure 3: Dose-response curves of lead ASOs. (A) Regression curves and absolute half maximal concentrations for all 16 lead ASOs were calculated using "log(agonist) vs. normalized response – Variable slope" (EC_{50}) and "log(inhibitor) vs. normalized response – Variable slope" (IC_{50}) functions in GraphPad Prism. *GBE1* WT transcript changes are plotted on the left Y-axis and *GBE1* MT transcript changes are on the right Y-axis.

A

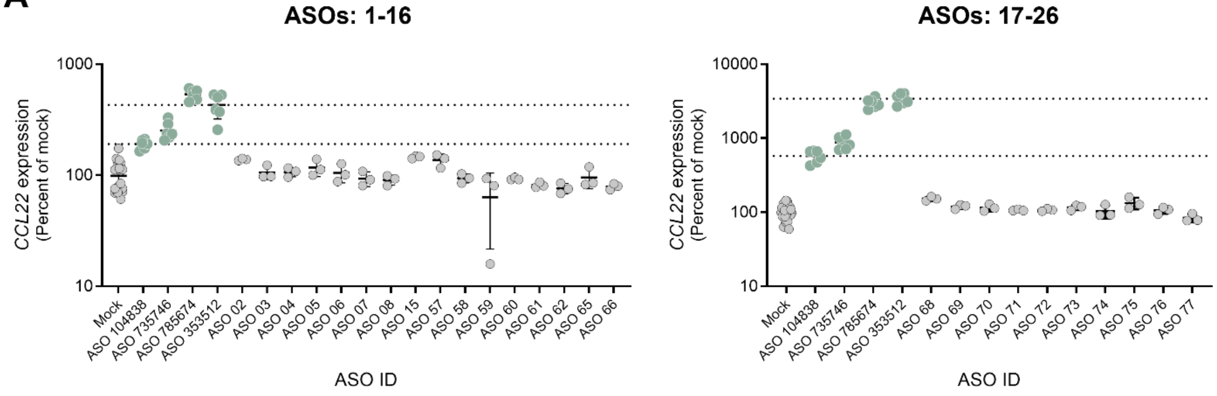

Supplementary Figure 4: None of the 26 ASOs tested in dose-response screen trigger innate immune response *in vitro*. (A) Measurement of *CCL22* expression by qRT-PCR in Bjab cells treated with the 26 ASOs at 1.6 uM for 24 hours. Bjab cells treated with known benchmark ASOs (ASO 10438, ASO 735746, ASO 785674 and ASO 353512) were used as controls in the assay. Total RNA level measured using Ribogreen was used to normalize target gene expression within each sample.

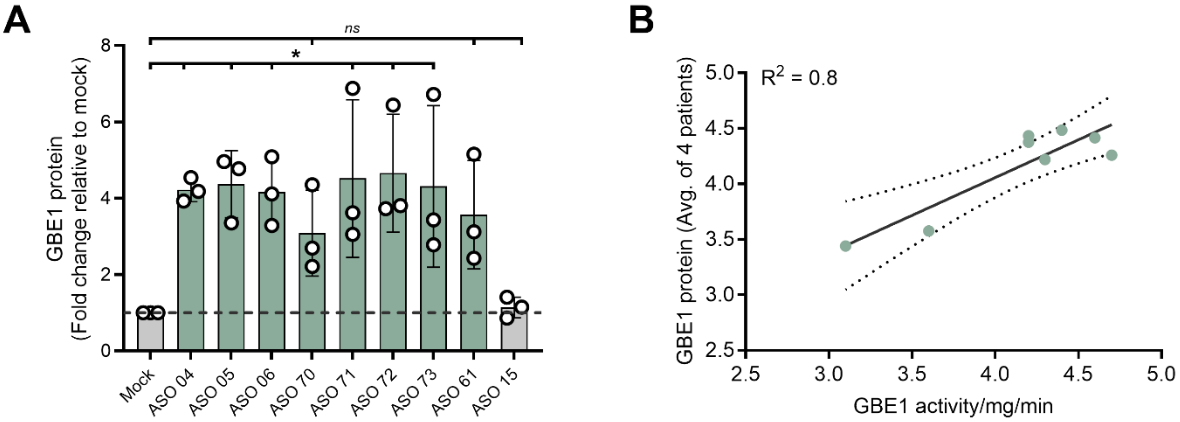

C

ASO ID	Perfect match	1 mismatch/gap	16 contiguous	1 mismatch/ gap genes
ASO 02	0	0	0	
ASO 03	0	0	0	
ASO 04	0	0	0	
ASO 05	0	0	0	
ASO 06	0	0	0	
ASO 07	0	0	0	
ASO 08	0	0	0	
ASO 60	0	0	0	
ASO 61	0	1	0	*ENSG00000289069*
ASO 68	0	0	0	
ASO 69	0	1	0	*TSPAN14*
ASO 70	0	0	0	
ASO 71	0	0	0	
ASO 72	0	1	0	*ZNF182*
ASO 73	0	3	0	*VXN, ZNF182, ENSG00000285791*
ASO 74	0	0	0	

Supplementary Figure 5: ASO-mediated rescue of GBE1 protein in additional patient cells and off-target analysis. (A) Quantitation of GBE1 protein levels in fibroblasts from patients 2, 3 and 4 treated with eight lead ASOs at 40 uM for 4 days. ASO 15 was included as a negative control (NC). The dashed line represents fold change equal to 1. Statistical analysis comparing the means of all treatment conditions to the Mock was performed using one-way ANOVA followed by Dunnett's post hoc analysis. ns, $p > 0.05$; *, $p < 0.05$. **(B)** Correlation between GBE1 protein (average of all four patient cells, **Fig. 3D**, **Suppl. Fig. 5A**) and enzyme activity levels in cells treated with eight lead ASOs. The dotted line in the correlation plot represent ± 95% confidence interval. **(C)** Predicted hybridization-dependent off-targets for the 16 lead ASOs.
